# Migration and Persistence of Human Influenza A Viruses, Vietnam, 2001–2008

**DOI:** 10.3201/eid1911.130349

**Published:** 2013-11

**Authors:** Mai Quynh Le, Ha Minh Lam, Vuong Duc Cuong, Tommy Tsan-Yuk Lam, Rebecca A Halpin, David E Wentworth, Nguyen Tran Hien, Le Thi Thanh, Hoang Vu Mai Phuong, Peter Horby, Maciej F. Boni

**Affiliations:** National Institute for Hygiene and Epidemiology, Hanoi, Vietnam (M.Q. Le, V.D. Cuong, N.T. Hien, L.T. Thanh, H.V.M. Phuong);; Oxford University Clinical Research Unit, Ho Chi Minh City, Vietnam (H.M. Lam, M.F. Boni);; University of Edinburgh, Edinburgh, Scotland, UK (H.M. Lam);; University of Oxford, Oxford, UK (T.T.-Y. Lam, P. Horby, M.F. Boni);; The J. Craig Venter Institute, Rockville, Maryland, USA (R.A. Halpin, D. E. Wentworth);; Oxford University Clinical Research Unit, Hanoi (P. Horby)

**Keywords:** influenza, tropics, migration, persistence, phylogeography, viruses, Vietnam

## Abstract

Understanding global influenza migration and persistence is crucial for vaccine strain selection. Using 240 new human influenza A virus whole genomes collected in Vietnam during 2001–2008, we looked for persistence patterns and migratory connections between Vietnam and other countries. We found that viruses in Vietnam migrate to and from China, Hong Kong, Taiwan, Cambodia, Japan, South Korea, and the United States. We attempted to reduce geographic bias by generating phylogenies subsampled at the year and country levels. However, migration events in these phylogenies were still driven by the presence or absence of sequence data, indicating that an epidemiologic study design that controls for prevalence is required for robust migration analysis. With whole-genome data, most migration events are not detectable from the phylogeny of the hemagglutinin segment alone, although general migratory relationships between Vietnam and other countries are visible in the hemagglutinin phylogeny. It is possible that virus lineages in Vietnam persisted for >1 year.

Understanding influenza dynamics in tropical regions is crucial for understanding global influenza epidemiology because dynamics between temperate and tropical regions are closely linked. Phylogenetic studies have supported eastern Asia, Southeast Asia, and the tropics as potential ecological sources of global influenza circulation (*1*,*2*), but others have suggested a variety of geographic regions as potential sources (*3*–*5*). Consequently, the role played by the tropics in the global epidemiology of influenza is still uncertain. Viral gene sequence data from tropical countries are crucial for understanding virus migratory routes within the tropics and between tropical and temperate countries.

Vietnam is an example of a tropical country that may play a major role in global influenza dynamics but for which relatively little is known about influenza epidemiology and genetic population structure of the viruses. Sentinel surveillance suggests that in Vietnam, influenza peaks 1–2 times per year, but neither the influenza-like illness (ILI) data nor the virologic confirmation data show a simple seasonal pattern; the trends for confirmed influenza cases fluctuate more than trends for ILI (*6*,*7*). Serologic studies indicate that annual influenza incidence in Vietnam is between 17% and 26% (*8*). The population of Vietnam is relatively young; according to contact patterns, most cases should occur among younger persons (*9*,*10*). Given Vietnam’s high population density and strong travel connections to eastern Asia, Southeast Asia, and Australia/New Zealand, Vietnam is as likely as any other country in eastern or Southeast Asia to support continuous, year-round circulation of a single influenza lineage (persistence) and potentially act as a global source of influenza viruses.

Previous global phylogenetic studies of influenza have demonstrated virus mixing globally (*3*,*4*,*11*), a lack of interseasonal persistence in temperate regions (*1*,*2*,*11*,*12*), and some evidence of persistence in subtropical regions (*5*,*13*). Time-series studies of confirmed influenza suggest (with exceptions [*14*]) that influenza does not exhibit the same strong and regular seasonality in tropical countries as it does in temperate zones (*15*–*19*) and that it could be constantly circulating throughout the year (*20*,*21*); however, in the latter 2 studies, phylogenetic analyses were not performed. We analyzed 240 newly sequenced influenza virus whole genomes from Vietnam, sampled through the Vietnam National Sentinel Surveillance System during 2001–2008 (*6*). We determined the relative strength of influenza migratory connections between Vietnam and the rest of the world, and we interpreted these results in the context of a sampling bias that seems to affect all sequence-based studies aiming for phylogeographic interpretations. On the basis of frequent sampling in 2007 and 2008, we assessed whether influenza viral lineages persisted in Vietnam during this period. However, we could not definitively conclude whether Vietnam represents a sink or a source population for influenza transmission.

Understanding global influenza migration and persistence patterns is crucial for maintaining a coordinated and efficient biannual strain selection process for influenza vaccine. Choices for future vaccine components will depend on recent availability of samples, and understanding each region’s contribution to global influenza circulation will help inform decisions based on viruses coming from highly connected or weakly connected regions.

## Methods

### Samples

During 2001–2008, as part of the Vietnam National Influenza Surveillance System, nasopharyngeal or throat swab samples were collected from patients seeking care for ILI at hospitals (*6*). Specimens were tested for influenza A and B viruses and were further subtyped for H1, H3, and H5 by reverse transcription PCR by using primers, probes, and reagents recommended by the Centers for Disease Control and Prevention and the World Health Organization (WHO). Samples that were positive for influenza A by PCR were selected for virus isolation, and isolates reaching a titer of 1:8 in hemagglutination assays were selected for sequencing analysis. All isolates were subtyped by using hemagglutination assays with reference antigens and antiserum from the WHO reagent kit. A total of 242 samples were shipped to the National Institutes of Health Influenza Genome Sequencing Project (USA) (*22*) for whole-genome sequencing at the J. Craig Venter Institute. Of the 242 samples, 2 were excluded from this analysis (1 that could not be sequenced and 1 from a patient with a mixed infection). The final dataset of the 240 whole-genome sequences comprised 145 influenza subtypes H3N2 and 95 H1N1 (GenBank accession nos. CY103972–CY105893). Table 1 shows the numbers and locations of the viruses.

**Table 1:** Influenza viruses successfully sequenced, Vietnam, 2001–2008

Virus subtype, geographic region	Year							
	2001	2002	2003	2004	2005	2006	2007	2008
H3N2								
Northern	1	0	4	18	4	0	66	0
Central	0	0	9	2	19	1	8	13
Southern	0	0	0	0	0	0	0	0
H1N1								
Northern	6	2	24	0	1	6	0	6
Central	0	0	1	0	2	4	0	36
Southern	0	0	0	0	0	6	0	1

### Datasets

For phylogeographic analysis, we compiled influenza virus sequences of subtypes H1N1 and H3N2 into 2 datasets: a regional dataset of whole-genome sequences from Asia and Australia/New Zealand and a global dataset of geographically subsampled sequences (50 replicates) of the hemagglutinin (HA) segment (Technical Appendix). For subsampling, we randomly sampled 12 sequences per geographic region per year.

### Phylogenetic Inference

Sequences were aligned by using the MUSCLE program, version 3.8 (*23*). Maximum-likelihood trees were inferred by using RAxML version 7.3.0 with 2,000 bootstrap replicates (*24*,*25*). For the regional HA datasets, phylogenetic trees with sampling date information were inferred by using BEAST version 1.6.2 (*26*) and a relaxed molecular clock (uncorrelated lognormal). The nucleotide substitution model was SRD06 + HKY85 + Γ, and the demographic models used were constant population size and Bayesian skyride (Technical Appendix).

### Analysis of Regional Migration

Migration analysis was conducted by using a straightforward parsimony method in the PAUP* program (*27*,*28*). The 2,000 bootstrap trees and the best maximum-likelihood tree inferred by RAxML were read into PAUP*, and nucleotide sequences were replaced by single-letter location codes assigned to a set of predefined global regions (Technical Appendix Figures 5, 6). Changes in location code were mapped onto the branches of the trees by using an ACCTRAN parsimony criterion (*28*). Analysis was performed on all segments to determine whether migration histories differed among them, which could have been caused by reassortment among influenza virus RNA segments. A strong reassortment signal was verified when standard mosaic and phylogenetic criteria were used (*29*) (Technical Appendix Figure 2).

### Analysis of Global Migration

For comparison, we performed the same migration analysis on the global dataset of 50 subsampled replicates for influenza virus subtypes H3N2 and H1N1. Migration matrices were built describing numbers of connections between 27 subtype H3N2 or 29 subtype H1N1 predefined geographic regions. We used Gephi software (*30*) to visualize the connections in the matrix. For regions with sufficient samples, we computed minimum distances to the trunk of the rooted phylogeny for all 50 subsampled trees (subtype H3N2 only) to determine whether viruses from different regions could be described as ancestral (close to the trunk) or derived (far from the trunk).

## Results

### Regional Migration of Influenza Virus (H3N2) HA

**Figure 1:**
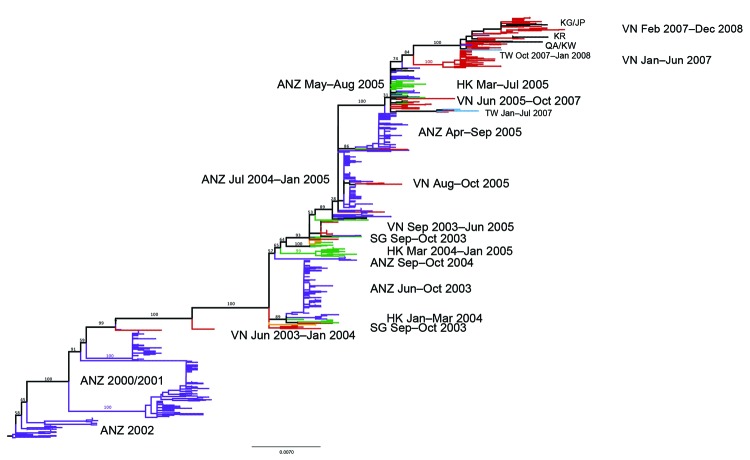
Maximum-likelihood phylogenetic tree (hemagglutinin segment) of the 787 sequences that comprise the regional influenza (H3N2) dataset. Tree is rooted on A/Canterbury/179/1999, and bootstrap values are shown on key nodes. Branches are colored by location: red, Vietnam; purple, Australia or New Zealand; green, Hong Kong; blue, Taiwan; orange, Singapore. Labels are shown directly to the left or right of the clades they are describing, with 2 exceptions: the label “VN Jun 2003–Jan 2004” refers to the viruses directly above it, and the label “ANZ 2000/2001” refers to the 2 major clades below it and above it. KG, Kyrgyzstan; VN, Vietnam; JP, Japan; KR, South Korea; QA, Qatar; KW, Kuwait; TW, Taiwan; HK, Hong Kong; ANZ, Australia and New Zealand. Scale bar indicates nucleotide substitutions per site.

The relationship between the subtype H3N2 HA sequences from Vietnam and other viruses sampled in the region is shown in Figure 1. Representative samples from Vietnam are available for 2003–2008 but not for 2006 when subtype H1N1 predominated. Viruses isolated in Vietnam show close relationships to viruses isolated in Hong Kong, Taiwan, Singapore, and Australia/New Zealand. Inferred from this tree were 20 parsimony-unambiguous migration events, 9 showing Vietnam–Hong Kong migration, 7 showing Vietnam–Australia/New Zealand migration, 2 showing Vietnam–Taiwan migration, 1 showing Vietnam–South Korea migration, and 1 showing Vietnam–Qatar migration. Clearly, because Hong Kong (68 sequences) and Australia/New Zealand (>500 sequences) were overrepresented in the regional dataset, most Vietnam migrations were associated with these 2 locations. Because we were initially uncertain how well a geographically regional phylogenetic tree would reflect the true migration patterns of influenza viruses in Vietnam, we performed a validation exercise to determine what proportion of global migration of influenza subtype H3N2 virus from Vietnam could be detected in a regional phylogenetic tree (Technical Appendix). Approximately 70% of Vietnam influenza migrations from a global analysis were also observed in the regional tree.

### Regional Migration of Influenza Virus (H1N1) HA

**Figure 2:**
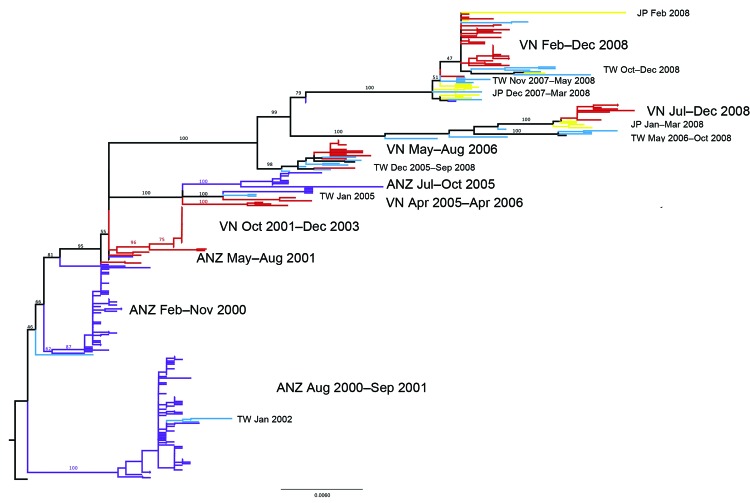
Maximum-likelihood phylogenetic tree (hemagglutinin segment) of the 300 sequences that comprise the regional influenza (H1N1) dataset. Tree is rooted on A/New Caledonia/20/1999, and bootstrap values are shown on key nodes. Branches are colored by location: red, Vietnam; purple, Australia or New Zealand; yellow, Japan; blue, Taiwan. Labels are shown directly to the left or right of the clades they are describing. JP, Japan; VN, Vietnam; TW, Taiwan; ANZ, Australia and New Zealand. Scale bar indicates nucleotide substitutions per site.

The relationship between influenza subtype H1N1 HA sequences from Vietnam and other regional viruses is shown in Figure 2. Representative samples from Vietnam are available for 2001–2008 but not for 2004 and 2007. Inferred from this tree are 10 parsimony-unambiguous migration events, 6 showing Vietnam–Taiwan migration, 2 showing Vietnam–Japan migration, and 2 showing Vietnam–Australia/New Zealand migration. As in the analysis for subtype H3N2, these migration links correspond with the viruses that were sequenced from the region during 2001–2008.

### Whole-Genome Migration Patterns

Because the regional trees included only sequences for which whole genomes were available, migration patterns were compared systematically across all 8 influenza segments. Because influenza viruses reassort, different event histories should be visible in phylogenies inferred separately for the 8 virus segments. Indeed, for the subtype H3N2 dataset, we observed a median of 14 parsimony-unambiguous migration events for the neuraminidase segment and a median of 41 for the matrix protein segment; the other segments fell somewhere in between (Table 2). Again, most migrations were with Australia/New Zealand and Hong Kong, indicating that the pattern of migration is similar across segments, although different numbers of migrations and different individual migration events are visible when different segments are analyzed. For the matrix protein and nonstructural protein segments of subtype H3N2 viruses, the large number of migration events may result from the larger number of topologically uncertain and polytomic nodes in these trees that had to be randomly resolved to compute the number of migration events; that is, sequences from one country could be mistakenly mixed with sequences from other countries, thus generating some artificial migration events in the parsimony analysis. The low confidence in the Vietnam–Singapore migration link for subtype H3N2 may result from the small number of whole-genome sequences available from Singapore, all of which were collected in 2003.

**Table 2:** Observed migration of influenza virus between Vietnam and other countries*

Virus subtype, country	Median no. events (95% range)							
	PB2	PB1	PA	HA	NP	NA	MP	NS
H3N2								
Aus/NZ	9 (5–13)	9 (5–13)	7 (4–10)	7 (3–10)	11 (6–16)	5 (2–8)	26 (19–33)	27 (18–36)
Hong Kong	4 (1–7)	4 (1–8)	6 (2–10)	5 (2–8)	6 (2–10)	7 (3.5–12)	12 (6–18)	5 (1–10.5)
Taiwan	2 (1–3)	3 (2–4)	3 (2–4)	3 (1–4)	2 (1–4)	1 (0–3)	3 (1–5)	2 (1–5)
Singapore	1 (0–3)	1 (0–3)	0 (0–2)	1 (0–3)	1 (0–6)	1 (0–3)	0 (0–2)	1 (0–3)
H1N1								
Taiwan	3 (1–6)	3 (1–5)	4 (2–7)	5 (2–7)	5 (2–8)	4 (2–8)	5 (2–10)	5 (2–8)
Aus/NZ	2 (0–5)	1 (0–4)	2 (0–5)	2 (0–5)	4 (1–8)	2 (0–4)	5 (2–9)	2 (0–6)
Japan	2 (1–4)	2 (1–4)	2 (1–4)	3 (1–4)	4 (1–8)	1 (0–2)	6 (2–9)	4 (2–7)

*Data from 2,000 bootstrapped trees for all 8 segments of regional datasets for influenza subtypes H3N2 and H1N1. PB, polymerase basic protein; PA, polymerase acidic protein; HA, hemagglutinin; NP, nucleocapsid protein; MP, matrix protein; NS, nonstructural protein; Aus/NZ, Australia/New Zealand.

For subtype H1N1 viruses, we observed 6–16 migration events across the trees inferred for the 8 segments (Table 2). In the bootstrapped data, the Vietnam–Taiwan and Vietnam–Japan migratory connections seem to be approximately equal, despite the fact that the best maximum-likelihood tree showed 6 Vietnam–Taiwan connections and 2 Vietnam–Japan connections. The migratory connection between Vietnam and Australia/New Zealand seems to be somewhat weaker, possibly because of substantially less sampling of Australia/New Zealand viruses in the subtype H1N1 dataset. In the subtype H1N1 dataset, the number of migration events for the matrix protein and nonstructural protein segments did not increase.

### Migration in Subsampled Global HA Trees

**Figure 3:**
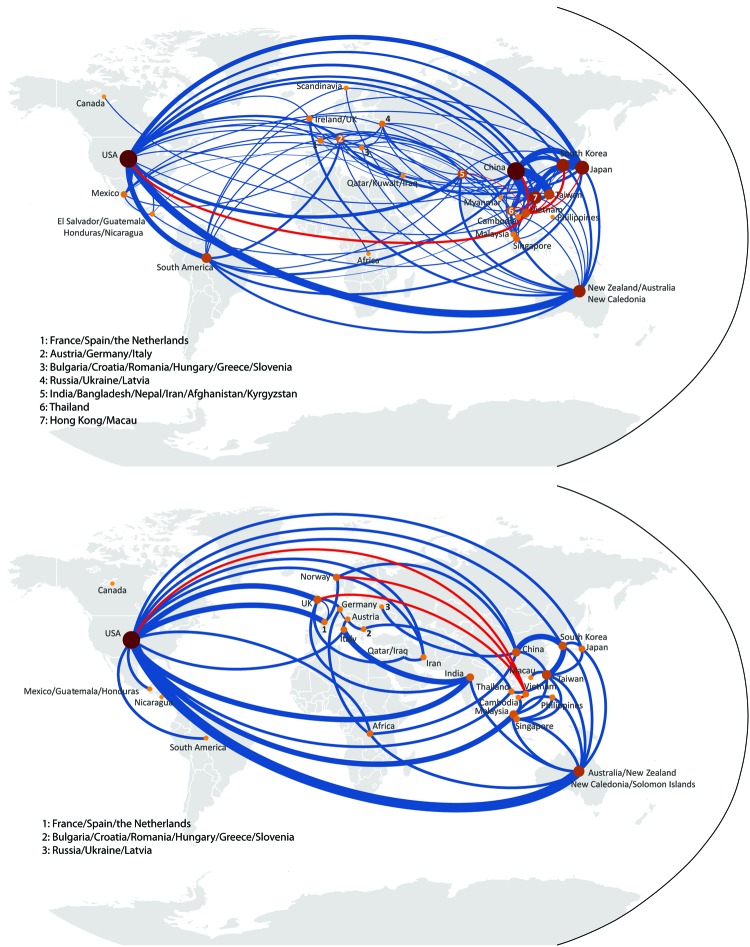
Global migration maps from fully subsampled global hemagglutinin tree for A) influenza (H3N2), based on 1,140 sequences, and B) influenza (H1N1), based on 554 sequences. The size and color of the nodes corresponds to the number of migration events associated with that location (median from 50 subsamples). The thickness of the lines corresponds to the number of migration events between 2 nodes. Red lines join Vietnam to other locations; blue lines join other locations. UK, United Kingdom; USA, United States.

Because it is clear that the presence and number of samples from different regions influence migration analysis, migratory patterns were reanalyzed on the global subsampled dataset to reduce the geographic bias present from having higher numbers of samples available from some regions than others. Using global HA trees for subtype H3N2 and H1N1 sequences, we constructed full migration matrices including all parsimony-unambiguous migration events among our predefined regions (Technical Appendix Figures 5, 6). These migration networks are shown in Figure 3, where the United States is a major hub of influenza migration and eastern Asia and Australia/New Zealand play major roles. The subtype H3N2 data show Vietnam connected with most other countries in eastern and Southeast Asia, with the United States, and weakly with southern Asia. The subtype H1N1 data show Vietnam connected with the United States and Europe but weakly with other Asian countries. For both subtypes, the total number of migration events associated with each node in the network is correlated with the number of samples available for that node (all p values were <10^−5^; Kendall and Spearman tests). Note that sample numbers are not identical for each node because for some regions <12 sequences per year were available, and these regions did not need to be subsampled for those years. Hence, undersampling and oversampling can generate this correlation. Despite our attempt to reduce geographic bias in the global dataset, inference on migration events is still closely associated with regional availability of samples; this bias appears to affect all phylogeographic studies.

**Figure 4:**
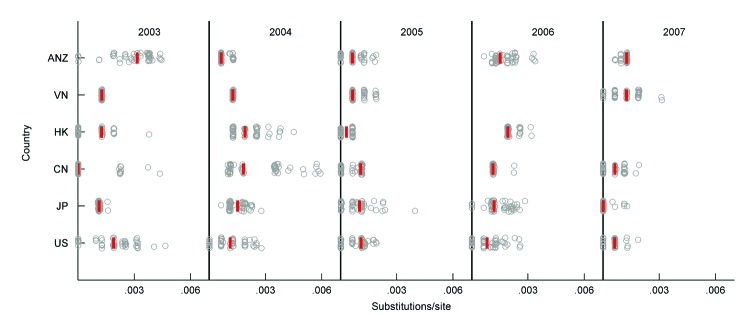
Minimum phylogenetic distance to the trunk, computed for the 50 subsampled global influenza (H3N2) phylogenies. Minimum distances are shown by year and by region, for 6 regions with sufficient sampling during 2003–2007. ANZ, Australia/New Zealand; VN, Vietnam; HK, Hong Kong; CN, China; JP, Japan; US, United States. Red lines show medians across 50 subsamples. For Vietnam in 2006 and Hong Kong in 2007, there were insufficient virus sequences.

For H3N2 sequences, to determine whether any region has the characteristics of an ecological source, we computed the phylogenetic distance of sequences from 6 well-sampled regions (China, Hong Kong, Japan, Vietnam, Australia/New Zealand, and the United States) to the trunk of the global maximum-likelihood phylogenetic tree (Figure 4). In 2003, for example, across all 50 subsampled trees, sequences isolated in China were typically closest to the trunk of the phylogenetic tree, indicating that these sequences are ancestral to other viral sequences sampled in 2003; this finding is consistent with the global replacement of subtype H3N2 viruses by the A/Fujian/411/2002-lineage that occurred in 2003. In general, for the years 2003–2007, in no region were sequences consistently ancestral, indicating that it is unlikely that there is a single global source of human influenza viruses. The more likely global migration model involves periodic global strain replacements originating in different regions in different years (*3*,*4*). There were not sufficient samples from all regions/years to perform this analysis on the subtype H1N1 dataset.

### Lineage Persistence

**Figure 5:**
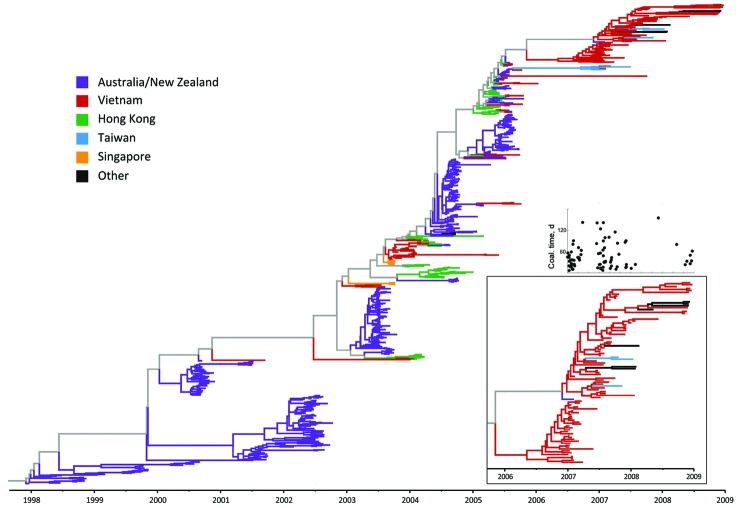
Maximum clade credibility tree for regional influenza (H3N2) hemagglutinin data, generated by BEAST version 1.6.2 (*26*) under a constant population model; these are the same sequences as shown on Figure 1, except 2 sequences from Vietnam were removed because of missing sampling dates (n = 785 sequences). Inset on the bottom right shows a magnification of the tree for the 2007–2008 Vietnam sequences, to highlight persistence during this time. The smaller inset above shows coalescent (coal.) times for the Vietnam sequences in the larger inset. The 2 *x*-axes on the insets coincide, and each black circle showing a coalescence time corresponds to the tip of a branch of Vietnam virus in the magnified-tree inset.

Figure 5 shows a Bayesian subtype H3N2 phylogenetic tree inferred from the time-stamped regional sequence data. The insets of this figure detail the 2007–2008 part of the phylogeny (87 sequences) and the coalescent times for the tips of these branches. It is difficult to draw a complete persistence picture for these viruses because of undersampling during the second quarter of 2007 and the first half of 2008 despite PCR-confirmed evidence of influenza virus activity during these periods (*6*). The median coalescent time for viruses from Vietnam sampled during this period is 37 days (interquartile range 21–72 days), and the insets in Figure 5 suggest that one of the lineages persisted in Vietnam for the 13 months from January 2007 through January 2008. An absence of samples from February through May 2008 makes it impossible to determine conclusively if this lineage persisted in Vietnam for the entire 2-year period.

To determine whether the lack of samples from other countries created an artifactual picture of lineage persistence in Vietnam during 2007–2008, we assembled a sequence set of all 672 viruses from Asia and Australia/New Zealand from 2006 through 2008. The maximum clade credibility tree of these sequences (Technical Appendix Figure 4) indicates that the Vietnam lineages separate into >10 distinct lineages when viewed in the context of all Asian/ Australia/New Zealand influenza viruses. One of these lineages persisted in Vietnam for 15 months (Technical Appendix Figure 1 panel A), and another persisted for 10–12 months (Technical Appendix Figure 1, panel B), suggesting that lineage persistence of ≥1 year may have occurred in Vietnam during 2007–2008. However, this type of analysis is very sensitive to phylogenetic uncertainty because the individual lineages (or subclades) contain few sequences and may not be robust to small changes in tree topology.

## Discussion

According to our analysis, the major migratory routes of influenza virus pass through the United States, eastern Asia, and Australia/New Zealand. Europe—despite its population density and consistency of wintertime influenza epidemics—was slightly less connected to other parts of the world when compared with the United States. These results are consistent with those of previous studies that showed eastern Asia (*2*) and tropical Asia (*1*) as key influenza source populations and the United States as a major contributing region (*3*). The new sequence data in this analysis support strong migratory connections between Vietnam and neighboring countries, the United States, and Europe. Our regional phylogenetic analysis supports a strong connection between Vietnam and Australia/New Zealand, but the global analysis reveals that Australia/New Zealand sequences are more closely related to sequences from Asian countries other than Vietnam. In addition, the inferred phylogenies provide evidence of virus persistence in Vietnam for >1 year. This is a major finding because strong migratory links and persistence are the 2 key features for a proposed source region for influenza transmission; long-term persistence in tropical regions may be associated with more antigenic evolution and immune escape if it can be shown that longer persistence gives the virus population more time to accumulate and fix antigenic changes (*2*,*31*,*32*).

In general, persistence analyses are difficult even with regular sequence sampling and weekly virologic confirmations. When attempting to assess the likelihood of influenza persistence in a focal region (e.g., Vietnam), we must sample outside the focal region to determine whether local viruses have been reintroduced from elsewhere. However, the more sampling in the nonfocal region, the more likely it becomes that we sample nonfocal viruses similar to focal viruses and that more diversity is detected in the nonfocal region, making it seem basal (closer to the root) to the focal region. There are no clear criteria for whether we have undersampled or oversampled the focal or the nonfocal region; thus, it is extremely difficult to state with certainty that an apparently local lineage has persisted in the same location. For the 2007–2008 Vietnam influenza sequences, viruses were sampled for most of this period and coalescence times were generally short, indicating that most of these viruses have a relatively recent ancestor in Vietnam. These data are consistent with and provide evidence for lineage persistence in Vietnam during this time. However, we know of no unbiased test that can reject the possibility of virus introduction. The perfect dataset for demonstrating lineage persistence would seem to be 52 viruses sampled in 52 weeks, with consecutive viruses differing at 0 or 1 nt positions.

A major limitation of all migration analyses performed with sequence data is geographic sampling bias: undersampling and oversampling. The more sequences that are available for a given location, the more likely it is that 1 of these sequences will be a recent immigrant, identifiable by the presence of similar sequences from other locations. To overcome this bias, subsampling is typically conducted (*3*,*5*) to ensure that the same numbers of sequences are used from each region. In the situation when too few sequences are available from a particular location, a smaller number of migratory links will be able to be inferred for that location. This second bias cannot be corrected with a subsampling strategy.

Our analysis of the global subsampled dataset showed that sample counts and strength of migratory connections were highly correlated. It has so far been impossible to determine the causal direction in this correlation. A migration signal can be weak because of a dearth of samples. Conversely, the small number of samples can be the result of low influenza activity and a corresponding weak migratory connection with other regions. The directionality of causation cannot be determined from sequence data alone. A sequence sampling strategy must be devised in the context of an influenza surveillance system, and the epidemiologic data and sequence data must be analyzed jointly. Disease prevalence and sequence data should be directly linked to provide a denominator to help determine whether undersampling or oversampling are truly occurring, which would allow for correction of sampling numbers across regions.

Despite this seemingly obvious point about oversampling, the counterpoint is that oversampling in influenza sequence data occurs with a high degree of pseudoreplication. Influenza sequence sampling in most scientific studies and public health contexts is conducted in such a way that each additional sequence sample is not an independent observation but, rather, is an observation with a high degree of correlation to recently collected samples (*33*). These pseudoreplicated samples should not, in principle, generate additional artificial migration events into the analysis because the dependency structure of the samples is entirely accounted for in the phylogeny. Nevertheless, a correlation between sample number and migration strength persists in the data, partially, at least, because a larger number of samples increases the probability that a distant recently introduced lineage is sampled.

New approaches are needed in order to fully account for all spatial, evolutionary, and epidemiologic dependencies in phylogeographic analyses. For recent phylogeographic studies, Bayesian approaches have been the method of choice (*1*,*3*,*4*,*34*–*37*), primarily because of their ability to account for uncertainty in evolutionary, demographic, and migratory parameters, but especially because of their ability to incorporate topological uncertainty into phylogenetic analyses. If these methods can be further developed to incorporate representativeness uncertainty—essentially, a prior distribution on the size of the sampling pool to account for the fact that some parts of the phylogeny will be oversampled while others will be undersampled—then this type of Bayesian analysis could serve as a powerful auxiliary tool in phylogeography, enabling us to determine whether sampling bias has a larger effect in some regions than others. Another role for Bayesian analysis of influenza sequences will be the application of Bayesian phylogeographic methods on whole-genome sequence data (*1*). For highly reassortant datasets, the presence of independent migration signals in 8 phylogenies (for the influenza virus 8 RNA segments) should act to reduce uncertainty for the inferred migration parameters.

We intended to elucidate the migratory pathways of influenza into and out of Vietnam and the likelihood of virus persistence in Vietnam. For each of these objectives, we recommend that future studies link phylogenetic analysis with prevalence data, allowing for correction of known biases and providing crucial complementary epidemiologic evidence for migration and persistence. If the source–sink framework is an oversimplification of global influenza circulation (*3*–*5*), Vietnam probably plays both roles on different occasions, given its close connections to other countries in Asia, Europe, and the United States.

Technical Appendix Supplementary methods.
