# Supplementary material for: Migration and Persistence of Human Influenza A Viruses, Vietnam, 2001–2008
Source: Emerg Infect Dis. 2013 Nov;19(11):1756–65. doi: 10.3201/eid1911.130349 (PMC3837676; doi:10.3201/eid1911.130349)
Supplement: Technical Appendix — Supplementary methods. [file 13-0349-Techapp-s1.pdf]

Article DOI: http://dx.doi.org/10.3201/eid1911.130349

Migration and Persistence of Human Influenza A viruses, Vietnam, 2001–2008

Technical Appendix

1. Supplementary methods

Primary Data Sets. For phylogeographic analysis, influenza virus sequences of both H1N1 and H3N2 subtypes were compiled into two datasets: a "regional data set" of whole-genome sequences from Asia and Australia/New Zealand (ANZ), and a "global data set" of geographically sub-sampled sequences (50 replicates) of the hemagglutinin (HA) segment. For the regional dataset, we downloaded from GenBank all influenza virus sequence data that met the following criteria: (a) the virus was isolated in one of Australia, New Zealand, or an Asian country, (b) all eight segments were sequenced, (c) the date of isolation was available, and (d) the virus was sampled between 1999 and 2008. Because Australian and New Zealand strains were very closely related to each other, with ancestry difficult to determine in many cases, we grouped these two countries into a single region. This regional dataset yielded 787 whole-genome sequences of subtype H3N2 and 300 whole-genome sequences of subtype H1N1 (including the new Vietnamese sequences). The regional dataset was assembled to have a dataset of manageable size on which we could perform phylogeographic analysis for the geographic region surrounding Vietnam. Australia and New Zealand were included in this analysis because of the known high frequency of travel between ANZ and Southeast Asia.

For the global HA datasets, we downloaded HA sequences from all countries, including only time-stamped sequences sampled between 2001 and 2008, with a minimum sequence length of 900nt. This yielded 3,934 H3N2 sequences and 1,970 H1N1 sequences. We generated 50 geographic subsamples of this dataset allowing for a maximum of 12 sequences per country per year. Viruses from the United States were subsampled with a maximum of three viruses per year from each of four geographic regions: Northeast, Southeast, Midwest, and West. Subsampled datasets comprised 1,140 H3N2 sequences and 554 H1N1 sequences.

Two additional datasets were assembled: one for validation of the regional dataset as representative of migration events for Vietnam (section 2 below), and one comprising all Asian and ANZ sequences from 2006 to 2008 to validate analyses on local persistence of influenza during this time (section 4.2 below).

Additional Data Sets. Two additional datasets were assembled: one for validation of the regional dataset as representative of migration events for Vietnam (section 2 below), and one comprising all Asian and ANZ sequences from 2006 to 2008 to validate analyses on local persistence of influenza during this time (section 4.2 below).

PAUP Migration Analysis. Migration events using the ACCTRAN parsimony criterion in PAUP* are reported as `unambiguous' or 'ambiguous'. Only unambiguous events where Vietnam was either the origin or the destination country were counted in the regional analysis, and all unambiguous events were counted in the global analysis to create a complete migration matrix among our pre-defined regions. In addition, PAUP* reports directionality for migration events, but we ignored this because it was not consistently repeatable across our analyses; all migration events described in this analysis should be interpreted as migratory connections, without certainty as to the direction of migration.

2. Validation of the regional dataset as a useful tool for inspecting migration events relating to Vietnam

As we were initially uncertain about the validity of using a 'regional tree' to infer migratory patterns for Vietnam, we constructed a partially sub-sampled global tree (PSGT), different from the 'global tree' mentioned in the main text, to determine what fraction of Vietnam-related global migration events were observable in the regional tree. The dataset for the PSGT was not restricted to Asia and ANZ and it was not restricted to include only whole genomes; it is partially subsampled because the Vietnam sequences are not subsampled but all other sequences are subsampled. The PSGT thus contains the exact same Vietnamese sequences as the regional tree and subsampled global sequences, from the same original sample set as in the `global tree' in the main paper. Hence, we can determine how many more migration events are associated with these VN sequences when we construct a global rather than regional phylogeny.

A total of 28 Vietnam-related parsimony-unambiguous migration events were inferred from the PSGT: five with China, four each with the United States and Hong Kong, three with Taiwan, and two each with Cambodia and South Korea. Single migration events were identified with Thailand, Myanmar, Japan, Iran, Qatar, France, Honduras, and Chile. Nineteen of these 28 migration events occurred between Vietnam and another country in East or Southeast Asia, indicating that roughly 70% of migration events for Vietnam can be observed by building a regional phylogenetic tree. Some of the long-range events present in the global tree (Honduras, Chile) must be exceptions as it is doubtful that there is a common or frequent migration route of influenza viruses between Vietnam and either of these countries.

Of the 20 regionally-inferred migration events (see main text), six could not be evaluated in the global tree, as the relevant sequences were not sub-sampled. Of the 14 remaining regionally-inferred migration events, all were supported in the global tree either directly, through an intermediary sequence, or through a missing ancestor in China; two VN-HK migrations and one VN-TW migration showed common ancestry in China with emigration (when sampling dates were taken into account) from China to both locations, indicating that the restricted sampling for the regional tree possibly missed the true migratory pattern in these situations. It is clear that some migration events will be missed by restricting sampling to a particular part of the world and to whole-genome sequences. Hence, in general, an individual migration event from a geographically regional tree should be viewed as a valid single observation — with probable, but not definite, phylogenetic support for the same migration event in a more complete global analysis — but the sum of these events does not represent a systematic migration analysis of influenza viruses among different countries. Rather, this type of regional migration analysis must be viewed as an investigation of influenza viruses in Vietnam and potential migration routes from/to New Zealand, Australia, Hong Kong, Singapore, and Taiwan — the only other countries with sufficient sampling in this dataset.

The migration events identified in the PSGT are listed in Table S1 below. Number of sequences and number of migration events were significantly correlated by Spearman ($p = 0.011$) and a Kendall ($p = 0.015$) tests.

Technical Appendix Table 1. Numbers of migration events between Vietnam and other countries/regions in the partially-subsampled global H3N2 tree.

	Number of Parsimony-Unambiguous Migration Events	Number of Sequences
China	5	84
USA	4	96
Hong Kong	4	72
Taiwan	3	53
Cambodia	2	26
South Korea	2	79
Thailand	1	51
Myanmar	1	17
Japan	1	89
Kyrgyzstan	1	5
Qatar	1	8
France	1	24
Central America	1	24
South America	1	48

3. Reassortment

Strong statistical evidence for reassortment was present in the datasets where whole genomes were available, i.e., the regional H3N2 and H1N1 datasets. The concatenated whole-genome datasets showed strong signals of mosaicism ($p < 10^{-11}$ for both, 3SEQ [1]), as did concatenated HA-NA datasets ($p < 10^{-9}$ for both, 3SEQ). Considering HA and NA for H3N2, a strong phylogenetic signal of reassortment was present (Technical Appendix Figure 2). As it is already known that human influenza viruses reassort frequently [2,3] this analysis was not pursued further.

4. Robustness of Persistence Result for Vietnamese sequences, 2007–2008

4.1 Bayesian skyride demographic model for H3N2 regional sequences

To determine if the viral persistence result described in the main text is robust to changes in viral effective population size, we performed phylogenetic inference with a Bayesian skyride model allowing for variable population size during the years 2001 to 2008. The main purpose of this analysis was to observe the presence/absence of population bottlenecks during 2007 and 2008 when viral lineages appeared to be persisting in Vietnam.

The MCMC was run for 150 million iterations, with the first 18 million designated as burn-in. Bayesian skyride was set as the demographic model, and other settings remained the same as for the constant population model. The effective sample size (ESS) values were 189 for the posterior, 34 for the prior, 93 for the likelihood, and 35 for the root height, which was the

best result that we observed after several runs and given the available computational time. The Maximum Clade Credibility (MCC) tree is shown as Technical Appendix Figure 3, with the bottom panel showing the Bayesian skyride plot with 95% posterior credible intervals. The natural-log Bayes factor for the constant population model over the skyride model was 23.7, but this unexpected result may be due to the incomplete convergence of the chain for the skyride analysis. The Bayesian skyride plot shows a drop in relative genetic diversity in the early part of 2008. This may be due to a real population bottleneck or an exogenous/endogenous strain replacement during this time.

In other analyses that we attempted for this dataset, we noted that inferred BSRs were not robust to various types of sub-analyses such as (*a*) sub-sampled regions, (*b*) individual datasets for separate regions, and (*c*) segments other than the hemagglutinin. For this reason, we were cautious in interpreting the BSRs as true reconstructions of past effective population size or relative genetic diversity, and we decided to present results in the main text based on the assumption of constant population size or constant genetic diversity.

4.2 Global sampling of sequences from 2006 through 2008

To determine if the viral persistence result described in the main text is robust to sampling frequencies of Asian and ANZ viruses during this time, we assembled a dataset with all Asian and ANZ hemagglutinin sequences (GenBank only) sampled between 2006 and 2008. In contrast to the migration analyses, it was not obvious how to devise a sub-sampling strategy for this type of analysis, where the goal is to find/reject evidence of lineage persistence in a focal region based on sampling in other regions. Hence, all sequences were included in the analysis. The final dataset included 672 H3N2 sequences, 88 of which were from Vietnam.

Two Bayesian phylogenetic trees were inferred with BEAST. The first was run under a constant population model, with a chain length of 100 million iterations and 10% burn-in (Technical Appendix Figure 4). The second was run under a Bayesian Skyride model, with a chain length of 100 million iterations and 45% burn-in.

Within the available computational time, the chain for the constant population model achieved near convergence in all parameters of interest (i.e., ESS >100; the ESS for the posterior was 183), while the BSR model did not converge. Therefore, we based our results on the maximum clade credibility tree of the constant population size model (Technical Appendix

Figure 4). This phylogeny shows that the 2007–2008 lineages from Figure 5 and Technical Appendix Figure 3 form more than a dozen sub-lineages when viewed in the regional context of all Asian and ANZ sequences. This was consistent in the incompletely converged BSR phylogeny. Two magnifications of the tree in Technical Appendix Figure 4 are shown in Technical Appendix Figure 1, suggesting lineage persistence in Vietnam for 15 months (panel A) and 11 months (panel B). These conclusions are sensitive to the absence of samples during certain periods, and to phylogenetic uncertainty that could affect the topology of these two subclades. Vietnam sentinel influenza data indicate that most weeks during this time period had positive influenza A/H3N2 cases; however, the last 2 months of 2007 saw an influenza B epidemic at which time there may have been no or low circulation of H3N2 viruses [4].

References

1. Boni MF, Posada D, Feldman MW (2007) An exact nonparametric method for inferring mosaic structure in sequence triplets. Genetics 176: 1035–47.

2. Holmes EC, Ghedin E, Miller N, Taylor J, Bao Y, et al. (2005) Whole-genome analysis of human influenza A virus reveals multiple persistent lineages and reassortment among recent H3N2 viruses. PLoS Biol 3: e300.

3. Nelson MI, Simonsen L, Viboud C, Miller MA, Taylor J, et al. (2006) Stochastic processes are key determinants of short-term evolution in influenza A virus. PLoS Pathog 2: e125.

4. Nguyen HT, Dharan NJ, Le MTQ, Nguyen NB, Nguyen CT, et al. (2009) National influenza surveillance in Vietnam, 2006–2007. Vaccine 28(2):398–402.

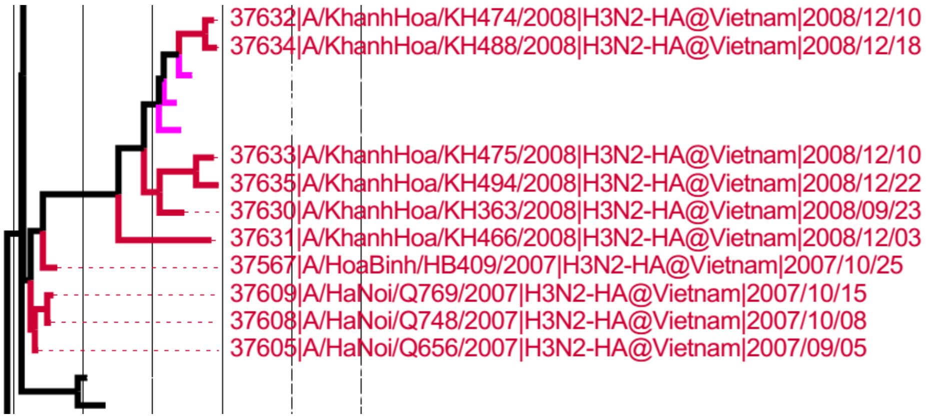

Technical Appendix Figure 1, panel A. Distance between adjacent vertical lines is 6 months. 15.5 months of persistence is seen in Vietnam, assuming intermediate sequences were also circulating in Vietnam. The three sequences at the pink tips are from Cambodia.

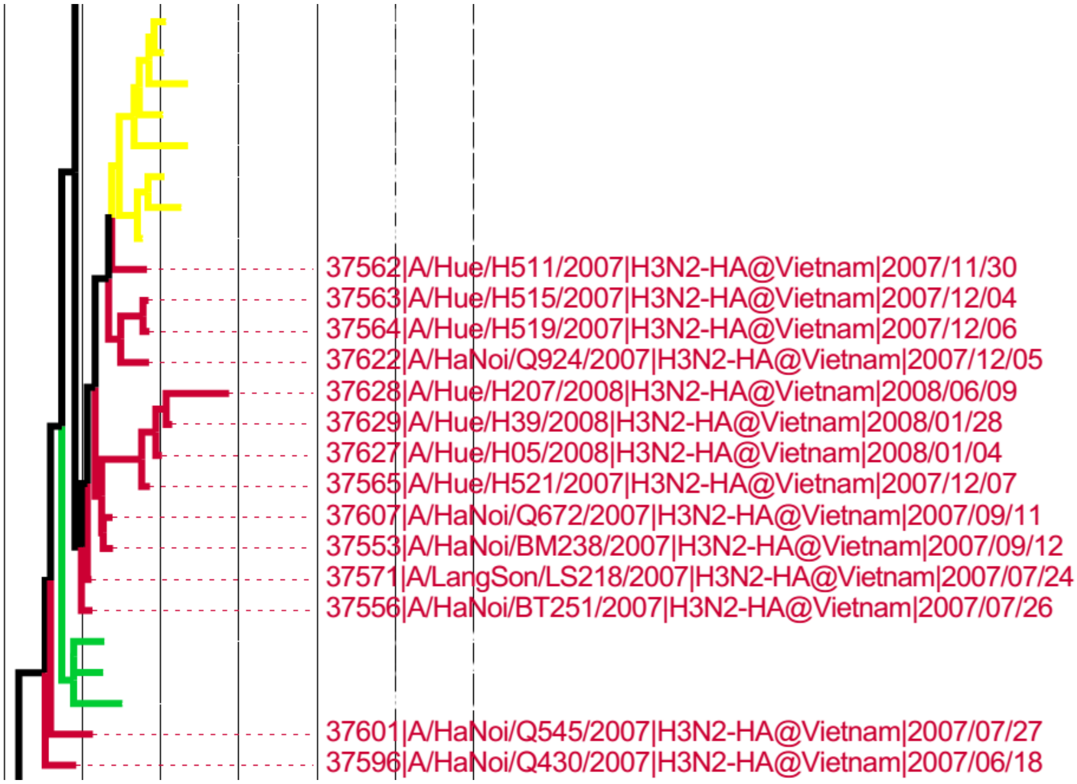

Technical Appendix Figure 1, panel B. Distance between adjacent vertical lines is 6 months. 10.5 to 11.5 months of persistence, depending on far down the phylogeny one looks. The three green tips represent sequences from China, two from Shanghai and one from Jiangxi province. The yellow clade at the top represents Japanese influenza viruses.

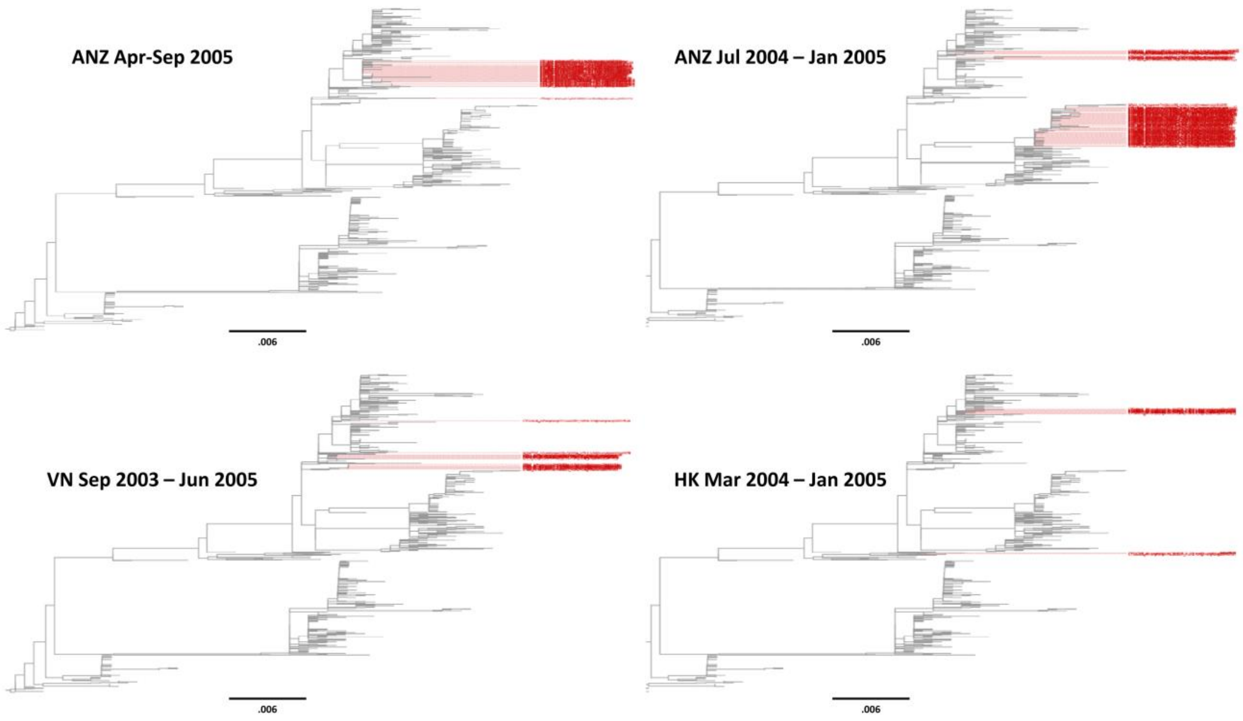

Technical Appendix Figure 2. Best ML trees (using RAxML) for the neuraminidase (NA) segment of the regional H3N2 dataset. From the HA tree of the regional dataset (Figure 1), four HA clades with strong reassortment signals were chosen and the sequences from these HA clades are shown in red here in the NA trees. The four clade labels in this figure correspond to the labels in Figure 1 of the main text. The NA sequences of the four chosen HA clades do not form monophyletic groups, indicating that the HA segments and NA segments of these viruses reassorted in the past.

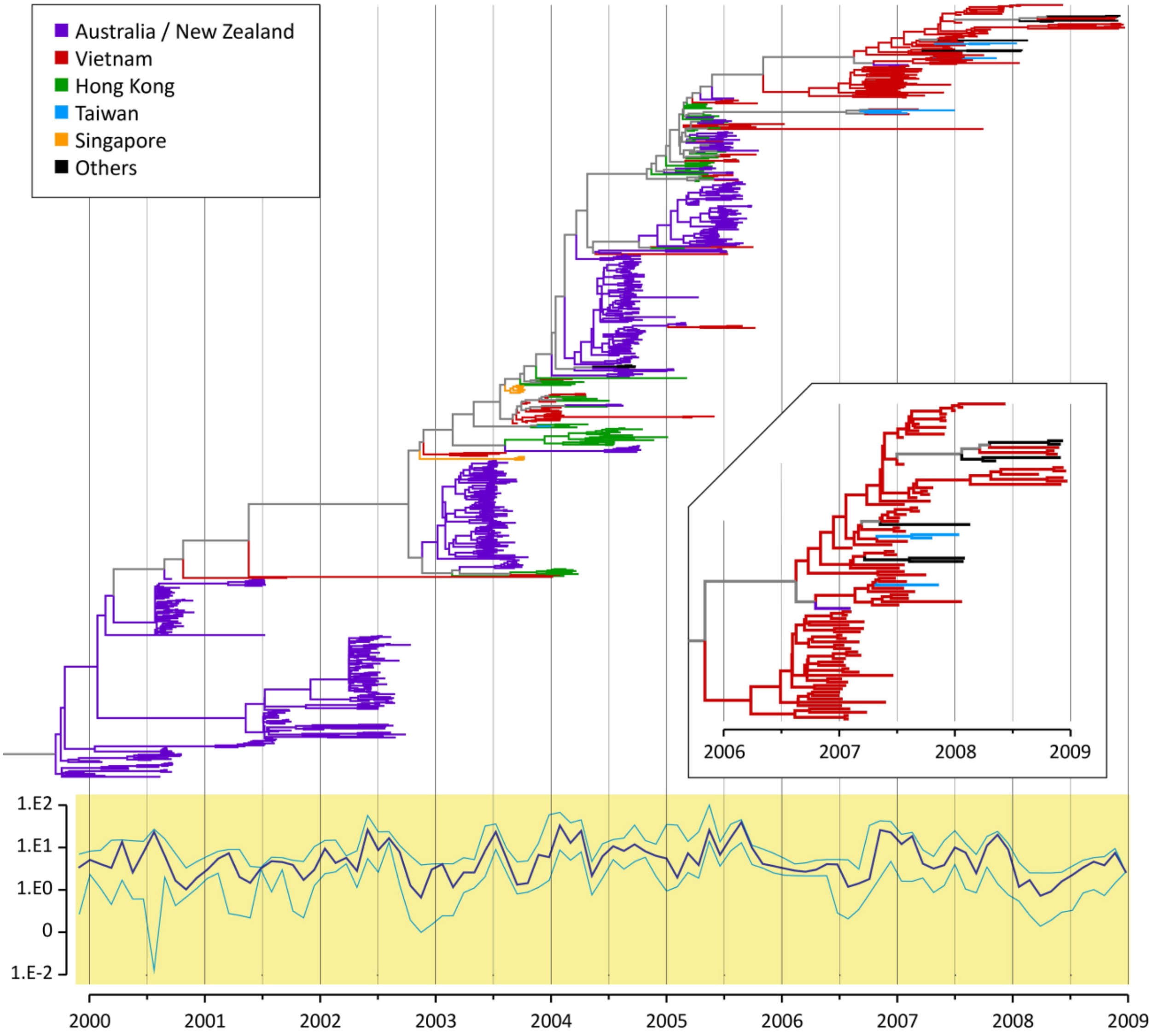

Technical Appendix Figure 3. Maximum clade credibility tree for regional H3N2 HA data, generated by BEAST under a Bayesian skyride population model; these are the same sequences as Figure 1 and Figure 5 of the main text. Inset on the right shows a magnification of the tree for the 2007–2008 Vietnam sequences, to show the persistence and lineage co-circulation patterns during this time. The panel at the bottom shows the effective population size/relative genetic diversity, i.e., the Bayesian skyride plot for the regional sequences from 2000 through 2008.

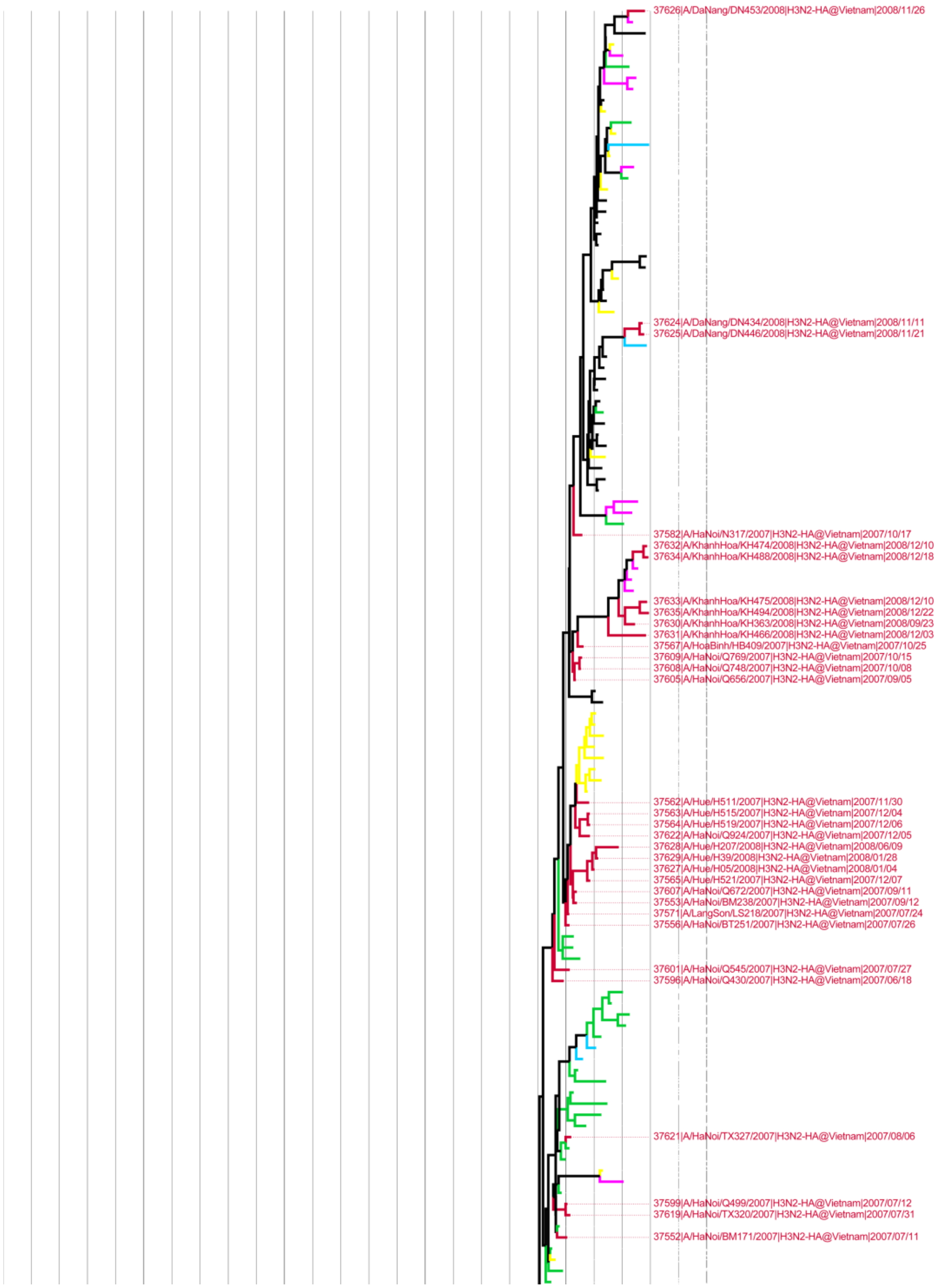

Technical Appendix Figure 4. Detail of maximum clade credibility tree for all Asian and ANZ H3N2 sequences sampled between 2006 and 2008, generated by BEAST under a constant population model. The tips of the tree are colored according to sampling location: Vietnam (red); Japan and Korea (yellow); China (green); Hong Kong (blue); Taiwan (turquoise); Singapore, Malaysia, and the Philippines (orange); Thailand, Cambodia, and Myanmar (pink); Australia, New Zealand, Solomon Islands, and New Caledonia (purple); all other countries shown in black. To view full tree, click on image.

	1	A	B	C	D	E	F	G	H	I	J	K	L	M	N	O	P	Q	R	S	T	U	V	W	X	Y	Z
1	0	0 (0-1)	1 (0-2)	2 (0-3)	1 (0-1)	0 (0-1)	1 (0-2)	0	0 (0-1)	1 (0-3)	2 (0-5)	0	1 (0-2)	0 (0-1)	2 (0-4)	2 (0-4)	0 (0-1)	0	0 (0-1)	0 (0-1)	0 (0-1)	4.5 (1-7)	0 (0-1)	0 (0-0)	0 (0-1)	0.5 (0-2)	0 (0-1)
A	0 (0-1)	0	0 (0-1)	0 (0-2)	0 (0-0)	0 (0-1)	0 (0-2)	0 (0-1)	0 (0-1)	0 (0-2)	0 (0-2)	0	0 (0-2)	0 (0-1)	0 (0-1)	0 (0-2)	0 (0-1)	0 (0-2)	0 (0-1)	0 (0-1)	0 (0-2)	1 (0-4)	0 (0-1)	0 (0-1)	0 (0-1)	0 (0-1)	0 (0-0)
B	1 (0-2)	0 (0-1)	0	0	0 (0-2)	0 (0-0)	0 (0-0)	0 (0-0)	0 (0-1)	1 (0-2)	0 (0-1)	0 (0-1)	0 (0-1)	0 (0-1)	1 (0-2)	0 (0-2)	0 (0-1)	0	1 (0-3)	0 (0-0)	0 (0-1)	0 (0-2)	0 (0-1)	0	0 (0-1)	0	0
C	2 (0-3)	0 (0-2)	0	0	1 (0-1)	0 (0-1)	0 (0-1)	1 (0-2)	7.5 (2-12	1 (0-3)	5 (2-9)	1 (0-2)	1 (0-2)	1 (0-3)	0 (0-2)	0 (0-2)	0 (0-1)	0 (0-1)	1 (0-2)	1 (0-3)	7.5 (3-11	2 (0-5)	2 (0-4)	1 (0-2)	3 (0-5)	3.5 (1-7)	0 (0-1)
D	1 (0-1)	0 (0-0)	0 (0-2)	1 (0-1)	0	0 (0-0)	0 (0-1)	0 (0-1)	0 (0-1)	0 (0-2)	0 (0-1)	0 (0-0)	0 (0-0)	0 (0-1)	1 (0-3)	0 (0-2)	0 (0-2)	0	0 (0-0)	0 (0-0)	0	0 (0-2)	0 (0-0)	0	0 (0-1)	0 (0-1)	0 (0-1)
E	0 (0-1)	0 (0-1)	0 (0-0)	0 (0-1)	0 (0-0)	0	0 (0-1)	0 (0-0)	0	0.5 (0-2)	0 (0-2)	0	0 (0-1)	0 (0-1)	0 (0-1)	0 (0-1)	0	0 (0-0)	0	0	0 (0-0)	1 (0-3)	0 (0-1)	0 (0-0)	0	1 (0-2)	1 (0-1)
F	1 (0-2)	0 (0-2)	0 (0-0)	0 (0-1)	0 (0-1)	0 (0-1)	0	0 (0-1)	0 (0-1)	1 (0-4)	0 (0-2)	0	0 (0-1)	0 (0-1)	0 (0-1)	1 (0-2)	0 (0-1)	0	1 (0-1)	0 (0-1)	0 (0-1)	1 (0-3)	0 (0-2)	0 (0-1)	1 (0-2)	0.5 (0-1)	0 (0-1)
G	0	0 (0-1)	0 (0-0)	1 (0-2)	0 (0-1)	0 (0-0)	0 (0-1)	0	0 (0-1)	0 (0-1)	0 (0-1)	0 (0-0)	0 (0-0)	1 (1-2)	1 (0-2)	0 (0-1)	0 (0-1)	0 (0-1)	0	0	0 (0-1)	1 (0-2)	2 (1-3)	0	0 (0-0)	0 (0-1)	0
H	0 (0-1)	0 (0-1)	0 (0-1)	7.5 (2-12	0 (0-1)	0	0 (0-1)	0 (0-1)	0	0 (0-1)	2 (0-5)	0 (0-1)	0 (0-1)	1 (0-2)	1 (0-3)	0 (0-2)	0 (0-2)	0	0 (0-1)	1 (0-4)	2 (0-5)	2 (0-4)	3 (0-6)	0 (0-0)	2 (0-4)	4 (1-6)	1 (0-2)
I	1 (0-3)	0 (0-2)	1 (0-2)	1 (0-3)	0 (0-2)	0.5 (0-2)	1 (0-4)	0 (0-1)	0 (0-1)	0	1 (0-3)	0	0 (0-1)	0 (0-1)	2 (0-3)	1 (0-3)	0	0 (0-2)	1 (1-3)	0	1 (0-2)	3 (0-6)	0.5 (0-2)	1 (0-2)	0 (0-2)	1 (0-3)	0
J	2 (0-5)	0 (0-2)	0 (0-1)	5 (2-9)	0 (0-1)	0 (0-2)	0 (0-2)	0 (0-1)	2 (0-5)	1 (0-3)	0	0 (0-0)	1 (0-2)	0 (0-1)	2 (0-4)	2 (1-5)	0 (0-2)	0 (0-2)	0 (0-2)	1 (0-5)	1 (0-4)	4 (2-9)	1 (0-3)	0 (0-2)	1 (0-3)	6 (2-11)	1 (0-3)
K	0	0	0 (0-1)	1 (0-2)	0 (0-0)	0	0	0 (0-0)	0 (0-1)	0	0 (0-0)	0	0	0 (0-0)	0 (0-1)	0	0 (0-0)	0	0	0	0 (0-1)	0 (0-2)	0	0	0 (0-1)	0 (0-0)	0
L	1 (0-2)	0 (0-2)	0 (0-1)	1 (0-2)	0 (0-0)	0 (0-1)	0 (0-1)	0 (0-0)	0 (0-1)	0 (0-1)	1 (0-2)	0	0	1 (0-2)	2 (1-4)	0 (0-1)	0 (0-1)	0	0 (0-1)	0 (0-1)	0 (0-1)	1 (0-2)	0	0	0	0 (0-0)	0 (0-0)
M	0 (0-1)	0 (0-1)	0 (0-1)	1 (0-3)	0 (0-1)	0 (0-1)	0 (0-1)	1 (1-2)	1 (0-2)	0 (0-1)	0 (0-1)	0 (0-0)	1 (0-2)	0	0 (0-1)	0 (0-1)	0 (0-1)	0	0 (0-0)	1 (0-2)	0 (0-2)	1 (0-2)	0 (0-2)	0	0 (0-2)	0 (0-1)	0
N	2 (0-4)	0 (0-1)	1 (0-2)	0 (0-2)	1 (0-3)	0 (0-1)	0 (0-1)	1 (0-2)	1 (0-3)	2 (0-3)	2 (0-4)	0 (0-1)	2 (1-4)	0 (0-1)	0	1 (0-3)	1 (0-3)	0	0 (0-1)	0 (0-2)	1 (0-3)	6.5 (3-9)	0 (0-2)	0 (0-0)	2 (0-3)	2 (0-4)	0 (0-0)
O	2 (0-4)	0 (0-2)	0 (0-2)	0 (0-2)	0 (0-2)	0 (0-1)	1 (0-2)	0 (0-1)	0 (0-2)	1 (0-3)	2 (1-5)	0	0 (0-1)	0 (0-1)	1 (0-3)	0	0 (0-0)	1 (0-2)	1 (0-3)	0 (0-1)	0 (0-1)	2 (1-5)	0 (0-0)	0 (0-0)	0 (0-1)	1 (0-2)	1 (0-1)
P	0 (0-1)	0 (0-1)	0 (0-1)	0 (0-1)	0 (0-2)	0	0 (0-1)	0 (0-1)	0 (0-2)	0	0 (0-2)	0 (0-0)	0 (0-1)	0 (0-1)	1 (0-3)	0 (0-0)	0	0	0	0 (0-1)	0 (0-1)	0 (0-1)	0	0	0 (0-1)	0 (0-1)	0 (0-0)
Q	0	0 (0-2)	0	0 (0-1)	0	0 (0-0)	0	0 (0-1)	0	0 (0-2)	0 (0-2)	0	0	0	0	1 (0-2)	0	0	0	0	0 (0-0)	1 (0-2)	0 (0-1)	0	0	0 (0-1)	1 (0-3)
R	0 (0-1)	0 (0-1)	1 (0-3)	1 (0-2)	0 (0-0)	0	1 (0-1)	0	0 (0-1)	1 (1-3)	0 (0-2)	0	0 (0-1)	0 (0-0)	0 (0-1)	1 (0-3)	0	0	0	1 (0-2)	1 (0-2)	0 (0-1)	0 (0-0)	0	1 (0-2)	0 (0-2)	0
S	0 (0-1)	0 (0-1)	0 (0-0)	1 (0-3)	0 (0-0)	0	0 (0-1)	0	1 (0-4)	0	1 (0-5)	0	0 (0-1)	1 (0-2)	0 (0-2)	0 (0-1)	0 (0-1)	0	1 (0-2)	0	0 (0-1)	1 (0-2)	0 (0-1)	0 (0-0)	1 (0-3)	1 (0-2)	0.5 (0-2)
T	0 (0-1)	0 (0-2)	0 (0-1)	7.5 (3-11	0	0 (0-0)	0 (0-1)	0 (0-1)	2 (0-5)	1 (0-2)	1 (0-4)	0 (0-1)	0 (0-1)	0 (0-2)	1 (0-3)	0 (0-1)	0 (0-1)	0 (0-0)	1 (0-2)	0 (0-1)	0	2.5 (0-5)	1 (0-2)	0 (0-1)	1 (0-3)	1 (0-4)	0 (0-0)
U	4.5 (1-7)	1 (0-4)	0 (0-2)	2 (0-5)	0 (0-2)	1 (0-3)	1 (0-3)	1 (0-2)	2 (0-4)	3 (0-6)	4 (2-9)	0 (0-2)	1 (0-2)	1 (0-2)	6.5 (3-9)	2 (1-5)	0 (0-1)	1 (0-2)	0 (0-1)	1 (0-2)	2.5 (0-5)	0	2 (0-4)	0 (0-1)	1 (0-3)	2 (0-5)	2 (1-4)
V	0 (0-1)	0 (0-1)	0 (0-1)	2 (0-4)	0 (0-0)	0 (0-1)	0 (0-2)	2 (1-3)	3 (0-6)	0.5 (0-2)	1 (0-3)	0	0	0 (0-2)	0 (0-2)	0 (0-0)	0	0 (0-1)	0 (0-0)	0 (0-1)	1 (0-2)	2 (0-4)	0	0 (0-1)	1 (0-2)	2 (1-3)	0
W	0 (0-0)	0 (0-1)	0	1 (0-2)	0	0 (0-0)	0 (0-1)	0	0 (0-0)	1 (0-2)	0 (0-2)	0	0	0	0 (0-0)	0 (0-0)	0	0	0	0 (0-0)	0 (0-1)	0 (0-1)	0 (0-1)	0	0	0 (0-1)	0
X	0 (0-1)	0 (0-1)	0 (0-1)	3 (0-5)	0 (0-1)	0	1 (0-2)	0 (0-0)	2 (0-4)	0 (0-2)	1 (0-3)	0 (0-1)	0	0 (0-2)	2 (0-3)	0 (0-1)	0 (0-1)	0	1 (0-2)	1 (0-3)	1 (0-3)	1 (0-3)	1 (0-2)	0	0	1 (0-2)	0
Y	0.5 (0-2)	0 (0-1)	0	3.5 (1-7)	0 (0-1)	1 (0-2)	0.5 (0-1)	0 (0-1)	4 (1-6)	1 (0-3)	6 (2-11)	0 (0-0)	0 (0-0)	0 (0-1)	2 (0-4)	1 (0-2)	0 (0-1)	0 (0-1)	0 (0-2)	1 (0-2)	1 (0-4)	2 (0-5)	2 (1-3)	0 (0-1)	1 (0-2)	0	0 (0-2)
Z	0 (0-1)	0 (0-0)	0	0 (0-1)	0 (0-1)	1 (0-1)	0 (0-1)	0	1 (0-2)	0	1 (0-3)	0	0 (0-0)	0	0 (0-0)	1 (0-1)	0 (0-0)	1 (0-3)	0	0.5 (0-2)	0 (0-0)	2 (1-4)	0	0	0	0 (0-2)	0

Technical Appendix Figure 5. Median number of migratory connections between regions for global H3N2 tree (95% ranges in parentheses). Based on 50 subsamples. Matrix is symmetric. A simple zero indicates that there were zero migration events between these two regions for all 50 subsamples. Region pairs where 95% range is bounded away from zero are shown in red. Region codes: 1, South America; A, Africa; B, Bulgaria, Croatia, Romania, Hungary, Greece, Slovenia; C, China; D, Denmark, Iceland, Norway, Sweden, Finland; E, El Salvador, Guatemala, Honduras, Nicaragua; F, France, Spain, Netherlands; G, Cambodia; H, Hong Kong, Macau; I, India, Bangladesh, Nepal, Iran, Afghanistan, Kyrgyzstan; J, Japan; K, Canada; L, Ireland, United Kingdom; M, Malaysia; N, New Zealand, Australia, New Caledonia; O, Austria, Germany, Italy; P, Philippines; Q, Qatar, Kuwait, Iraq; R, Russia, Ukraine, Latvia; S, Singapore; T, Taiwan; U, USA; V, Vietnam; W, Myanmar; X, Thailand; Y, South Korea, North Korea; Z, Mexico.

	1	2	3	A	B	C	D	E	F	G	H	I	J	K	L	M	N	O	P	Q	R	S	T	U	V	W	X	Y	Z
1	0	0	0	0 (0-1)	0	0	1 (0-1)	0	0 (0-1)	0	0	0 (0-1)	0 (0-1)	0	0 (0-1)	0	0 (0-1)	0	0	1 (0-1)	0	0	0	1 (0-3)	0	0	0	0	0
2	0	0	0 (0-0)	1 (0-1)	0.5 (0-2	0	0 (0-1)	0 (0-1)	0 (0-1)	0	0	0 (0-1)	0 (0-0)	0 (0-0)	1 (0-2)	0	0 (0-1)	0 (0-1)	0	0 (0-1)	0 (0-1)	0	0 (0-1)	0 (0-1)	0 (0-1)	0	0	0	0
3	0	0 (0-0)	0	0 (0-1)	0 (0-1)	0	0 (0-1)	0 (0-1)	0 (0-1)	0	0	2 (1-3)	0 (0-0)	0 (0-2)	0	0 (0-0)	0 (0-1)	1 (0-3)	0	0 (0-0)	0 (0-1)	0	0 (0-1)	1 (0-2)	0 (0-1)	0	0	0	0 (0-0)
A	0 (0-1)	1 (0-1)	0 (0-1)	0	0 (0-1)	1 (0-1)	0	0	0 (0-1)	0	0	0 (0-1)	0 (0-1)	0	0	0	1 (0-2)	0	0	0 (0-0)	0	0	0 (0-1)	1 (0-2)	0 (0-1)	0	0	0 (0-0)	0
B	0	0.5 (0-2	0 (0-1)	0 (0-1)	0	0	0 (0-1)	0 (0-1)	0 (0-2)	0	0 (0-0)	0 (0-0)	0 (0-0)	0 (0-1)	0 (0-0)	0	0 (0-0)	1 (0-3)	0	0 (0-1)	0 (0-1)	0	0	0 (0-1)	0 (0-1)	0 (0-0)	1 (0-1)	0 (0-0)	0 (0-1)
C	0	0	0	1 (0-1)	0	0	1 (0-2)	0	0	0	0 (0-1)	0	0 (0-1)	0	0 (0-0)	0	0 (0-1)	0	0 (0-1)	0	1 (1-1)	0	1 (1-2)	1 (0-3)	0 (0-0)	0	0	2 (0-3)	0
D	1 (0-1)	0 (0-1)	0 (0-1)	0	0 (0-1)	1 (0-2)	0	0 (0-0)	1 (1-2)	0	0 (0-1)	0 (0-0)	0 (0-1)	0 (0-1)	0 (0-1)	0 (0-0)	0 (0-2)	0 (0-1)	0	0 (0-0)	0 (0-0)	0	0 (0-1)	1 (0-4)	1 (0-2)	0	0	0 (0-1)	0 (0-1)
E	0	0 (0-1)	0 (0-1)	0	0 (0-1)	0	0 (0-0)	0	0	0	0	0 (0-0)	0 (0-1)	0 (0-1)	0	0	0 (0-0)	0 (0-1)	0	0 (0-1)	0	0	0 (0-1)	0 (0-2)	0 (0-1)	0	0	0 (0-1)	0 (0-1)
F	0 (0-1)	0 (0-1)	0 (0-1)	0 (0-1)	0 (0-2)	0	1 (1-2)	0	0	0	0	0 (0-1)	0 (0-1)	0	0.5 (0-1	0	0 (0-2)	0 (0-1)	0 (0-0)	0 (0-1)	0 (0-1)	0	0 (0-0)	2 (1-2)	0 (0-0)	0 (0-1)	0 (0-1)	0 (0-1)	0 (0-1)
G	0	0	0	0	0	0	0	0	0	0	0	0	0 (0-1)	0	0	1 (0-1)	0 (0-1)	0	0	0	0	0	0 (0-1)	0 (0-1)	1 (0-2)	0	0	0	0
H	0	0	0	0	0 (0-0)	0 (0-1)	0 (0-1)	0	0	0	0	0	0	0	0	0 (0-0)	0 (0-1)	0 (0-1)	0	0	0	0	1 (0-2)	0 (0-1)	0	0	0 (0-1)	0 (0-1)	0
I	0 (0-1)	0 (0-1)	2 (1-3)	0 (0-1)	0 (0-0)	0	0 (0-0)	0 (0-0)	0 (0-1)	0	0	0	0 (0-1)	0 (0-2)	1 (0-2)	0 (0-2)	1 (0-3)	0 (0-1)	0 (0-1)	0 (0-1)	0	0	0 (0-1)	2 (0-5)	0 (0-0)	0	0	0	0 (0-1)
J	0 (0-1)	0 (0-0)	0 (0-0)	0 (0-1)	0 (0-0)	0 (0-1)	0 (0-1)	0 (0-1)	0 (0-1)	0 (0-1)	0	0 (0-1)	0	0 (0-1)	0 (0-1)	0 (0-1)	1 (0-2)	0 (0-2)	0	0 (0-0)	0	0	1 (0-3)	1 (0-3)	0 (0-1)	0	0 (0-0)	1 (0-3)	0 (0-1)
K	0	0 (0-0)	0 (0-2)	0	0 (0-1)	0	0 (0-1)	0 (0-1)	0	0	0	0 (0-2)	0 (0-1)	0	0 (0-1)	0 (0-1)	0 (0-1)	0 (0-1)	0 (0-1)	0	0	0	0 (0-1)	0 (0-1)	0	0 (0-0)	0	0	0 (0-1)
L	0 (0-1)	1 (0-2)	0	0	0 (0-0)	0 (0-0)	0 (0-1)	0	0.5 (0-1	0	0	1 (0-2)	0 (0-1)	0 (0-1)	0	0 (0-0)	0 (0-1)	0 (0-1)	0	1 (0-1)	0 (0-1)	0	0 (0-0)	2 (1-4)	1 (0-2)	0 (0-0)	0	0 (0-0)	0
M	0	0	0 (0-0)	0	0	0	0 (0-0)	0	0	1 (0-1)	0 (0-0)	0 (0-2)	0 (0-1)	0 (0-1)	0 (0-0)	0	1 (0-2)	0	1 (0-3)	0	0	1 (0-3)	1 (0-2)	2 (0-4)	0 (0-1)	0 (0-0)	0 (0-1)	0	0
N	0 (0-1)	0 (0-1)	0 (0-1)	1 (0-2)	0 (0-0)	0 (0-1)	0 (0-2)	0 (0-0)	0 (0-2)	0 (0-1)	0 (0-1)	1 (0-3)	1 (0-2)	0 (0-1)	0 (0-1)	1 (0-2)	0	0 (0-1)	1 (0-2)	0 (0-1)	0	1 (0-2)	1 (0-3)	3.5 (2-6	0 (0-1)	0 (0-1)	0 (0-0)	1 (0-2)	0 (0-1)
O	0	0 (0-1)	1 (0-3)	0	1 (0-3)	0	0 (0-1)	0 (0-1)	0 (0-1)	0	0 (0-1)	0 (0-1)	0 (0-2)	0 (0-1)	0 (0-1)	0	0 (0-1)	0	0	0 (0-1)	0 (0-0)	0	0 (0-1)	0 (0-1)	0 (0-1)	0	0 (0-1)	0 (0-1)	0 (0-0)
P	0	0	0	0	0	0 (0-1)	0	0	0 (0-0)	0	0	0 (0-1)	0	0 (0-1)	0	1 (0-3)	1 (0-2)	0	0	0	0	1 (0-2)	0 (0-1)	0 (0-1)	0	0	0	0 (0-1)	0
Q	1 (0-1)	0 (0-1)	0 (0-0)	0 (0-0)	0 (0-1)	0	0 (0-0)	0 (0-1)	0 (0-1)	0	0	0 (0-1)	0 (0-0)	0	1 (0-1)	0	0 (0-1)	0 (0-1)	0	0	0	0	0 (0-0)	0 (0-1)	0 (0-1)	0	0	0	0 (0-0)
R	0	0 (0-1)	0 (0-1)	0	0 (0-1)	1 (1-1)	0 (0-0)	0	0 (0-1)	0	0	0	0	0	0 (0-1)	0	0	0 (0-0)	0	0	0	0	0	0 (0-1)	0	0	0	0 (0-1)	0
S	0	0	0	0	0	0	0	0	0	0	0	0	0	0	0	1 (0-3)	1 (0-2)	0	1 (0-2)	0	0	0	1 (0-1)	0 (0-1)	0 (0-0)	0	0	0 (0-0)	0
T	0	0 (0-1)	0 (0-1)	0 (0-1)	0	1 (1-2)	0 (0-1)	0 (0-1)	0 (0-0)	0 (0-1)	1 (0-2)	0 (0-1)	1 (0-3)	0 (0-1)	0 (0-0)	1 (0-2)	1 (0-3)	0 (0-1)	0 (0-1)	0 (0-0)	0	1 (0-1)	0	0 (0-1)	0 (0-1)	0	1 (0-1)	2 (0-4)	0 (0-0)
U	1 (0-3)	0 (0-1)	1 (0-2)	1 (0-2)	0 (0-1)	1 (0-3)	1 (0-4)	0 (0-2)	2 (1-2)	0 (0-1)	0 (0-1)	2 (0-5)	1 (0-3)	0 (0-1)	2 (1-4)	2 (0-4)	3.5 (2-6	0 (0-1)	0 (0-1)	0 (0-1)	0 (0-1)	0 (0-1)	0 (0-1)	0	1 (0-3)	1 (0-2)	1 (0-2)	1 (0-2)	1 (0-3)
V	0	0 (0-1)	0 (0-1)	0 (0-1)	0 (0-1)	0 (0-0)	1 (0-2)	0 (0-1)	0 (0-0)	1 (0-2)	0	0 (0-0)	0 (0-1)	0	1 (0-2)	0 (0-1)	0 (0-1)	0 (0-1)	0	0 (0-1)	0	0 (0-0)	0 (0-1)	1 (0-3)	0	0 (0-0)	0 (0-1)	0 (0-2)	0 (0-1)
W	0	0	0	0	0 (0-0)	0	0	0	0 (0-1)	0	0	0	0	0 (0-0)	0 (0-0)	0 (0-0)	0 (0-1)	0	0	0	0	0	0	1 (0-2)	0 (0-0)	0	0	0 (0-0)	0
X	0	0	0	0	1 (0-1)	0	0	0	0 (0-1)	0	0 (0-1)	0	0 (0-0)	0	0	0 (0-1)	0 (0-0)	0 (0-1)	0	0	0	0	1 (0-1)	1 (0-2)	0 (0-1)	0	0	0	0
Y	0	0	0	0 (0-0)	0 (0-0)	2 (0-3)	0 (0-1)	0 (0-1)	0 (0-1)	0	0 (0-1)	0	1 (0-3)	0	0 (0-0)	0	1 (0-2)	0 (0-1)	0 (0-1)	0	0 (0-1)	0 (0-0)	2 (0-4)	1 (0-2)	0 (0-2)	0 (0-0)	0	0	0
Z	0	0	0 (0-0)	0	0 (0-1)	0	0 (0-1)	0 (0-1)	0 (0-1)	0	0	0 (0-1)	0 (0-1)	0 (0-1)	0	0	0 (0-1)	0 (0-0)	0	0 (0-0)	0	0	0 (0-0)	1 (0-3)	0 (0-1)	0	0	0	0

Technical Appendix Figure 6. Median number of migratory connections between regions for global H1N1 tree (95% ranges in parentheses). Based on 50 subsamples. Matrix is symmetric. A simple zero indicates

that there were zero migration events between these two regions for all 50 subsamples. Region pairs where 95% range is bounded away from zero are shown in red. Region codes: 1, South America; A, Africa; B, Bulgaria, Croatia, Romania, Hungary, Greece, Slovenia; C, China; D, Denmark, Iceland, Norway, Sweden, Finland; E, El Salvador, Guatemala, Honduras, Nicaragua; F, France, Spain, Netherlands; G, Cambodia; H, Hong Kong, Macau; I, India, Bangladesh, Nepal, Iran, Afghanistan, Kyrgyzstan; J, Japan; K, Canada; L, Ireland, United Kingdom; M, Malaysia; N, New Zealand, Australia, New Caledonia; O, Austria, Germany, Italy; P, Philippines; Q, Qatar, Kuwait, Iraq; R, Russia, Ukraine, Latvia; S, Singapore; T, Taiwan; U, USA; V, Vietnam; W, Myanmar; X, Thailand; Y, South Korea, North Korea; Z, Mexico.
